# A qualitative exploration of the psychosocial needs of people living with long‐term conditions and their perspectives on online peer support

**DOI:** 10.1111/hex.13814

**Published:** 2023-07-17

**Authors:** Hannah Rowlands, Elly Aylwin‐Foster, Matthew Hotopf, Lauren Rayner, Alan Simpson, Grace Lavelle, Vanessa Lawrence

**Affiliations:** ^1^ Department of Psychological Medicine, Institute of Psychiatry, Psychology and Neuroscience King's College London London UK; ^2^ Florence Nightingale Faculty of Nursing, Midwifery and Palliative Care King's College London London UK; ^3^ Health Service and Population Research, Institute of Psychiatry, Psychology and Neuroscience King's College London London UK

**Keywords:** long‐term conditions, mental health, peer support, physical health, psychosocial

## Abstract

**Introduction:**

Approximately 20% of people with a long‐term condition (LTC) experience depressive symptoms (subthreshold depression [SUBD]). People with SUBD experience depressive symptoms that do not meet the diagnostic criteria for major depressive disorder. However, there is currently no targeted psychological support for people with LTCs also experiencing SUBD. Online peer support is accessible, inexpensive and scalable, and might offer a way of bridging the gap in psychosocial care for LTC patients. This article explores the psychosocial needs of people living with LTCs and investigates their perspectives on online peer support interventions to inform their future design.

**Methods:**

Through a co‐produced participatory approach, online focus groups were completed with people with lived experience of LTCs. Focus groups were audio recorded and transcribed verbatim. Reflexive thematic analysis (TA) was conducted adopting a critical‐realist approach and an inductive analysis methodology that sought to follow participants' priorities and concerns.

**Results:**

Ten people with a range of LTCs participated across three online focus groups, lasting an average of 95 (±10.1) min. The mean age was 57 (±11.4) years and 60% of participants identified as female. The three key emerging themes were: (1) relationship between self and outside world; (2) past experiences of peer support; and (3) philosophy and vision of peer support. Adults living with LTCs shared their past experiences of peer support and explored their perspectives on how future online peer support platforms may support their psychosocial needs.

**Conclusion:**

Despite the negative impact(s) of having a long‐term physical health condition on mental health, physical and mental healthcare are often treated as separate entities. The need for an integrated approach for people with LTCs was clear. Implementation of online peer support to bridge this gap was supported, but there was a clear consensus that these interventions need to be co‐produced and carefully designed to ensure they feel safe and not commercialised or prescriptive. Shared explorations of the potential benefits and concerns of these online spaces can shape the philosophy and vision of future platforms.

**Patient or Public Contribution:**

This work is set within a wider project which is developing an online peer support platform for those living with LTCs. A participatory, co‐produced approach is integral to this work. The initial vision was steered by the experiences of our Patient and Public Involvement (PPI) groups, who emphasised the therapeutic value of peer‐to‐peer interaction. The focus groups confirmed the importance and potential benefit of this project. This paper represents the perspectives of PPI members who collaborate on research and public engagement at the mental–physical interface. A separate, independent Research Advisory Group (RAG), formed of members also living with LTCs, co‐produced study documents, topic guides, and informed key decision‐making processes. Finally, our co‐investigator with lived experience (E. A.F.) undertook the analysis and write‐up alongside colleagues, further strengthening the interpretation and resonance of our work. She shares first joint authorship, and as a core member of the research team, ensures that the conduct of the study is firmly grounded in the experience of people living with LTCs.

## 1 INTRODUCTION

An estimated 15 million people in England live with a long‐term condition (LTC).^1^ Of these people, approximately 20% screen positive for major depressive disorder (MDD) as defined by the DSM‐5 (Diagnostic and Statistical Manual of Mental Disorders, Fifth Edition) and a further 20% experience subthreshold depression (SUBD).^2^, ^3^ SUBD is the experience of depressive symptoms that do not meet the diagnostic criteria for MDD.^4^, ^5^ In those with LTCs, SUBD is associated with having a significant impact on people's lives, including reduced quality of life, poorer reported physical health outcomes and increased risk of MDD.^5^, ^6^, ^7^ SUBD is also a key risk factor for major depression, with 42% of patients who have SUBD comorbid with type 2 diabetes or heart disease developing major depression within 2 years.^7^, ^8^ Currently, there is no targeted psychological support for people with LTCs who are also experiencing SUBD. To prevent the escalation to MDD, the needs of those with LTCs experiencing SUBD need to be more carefully understood.

Online health interventions reportedly increase self‐management behaviours and improve wellbeing.^9^, ^10^ Studies in patients with LTCs have highlighted improved self‐efficacy, adaptive coping and empowerment as benefits of participating in online support groups.^11^ Peer support is defined as ‘a range of approaches through which people with similar LTCs or experiences support each other to better understand the condition and aid recovery or self‐management’.^12^ Peer support may take place face‐to‐face, over the phone or online.^13^ Online peer support platforms often embed a psychoeducation element. Psychoeducation interventions are defined as a ‘professionally delivered treatment modality that integrates and synergizes psychotherapeutic and educational interventions’^14^ and are considered more holistic than traditional medical model interventions.^14^ However, there is currently little evidence exploring the effectiveness of online peer support combined with psychoeducation interventions to support people with LTCs experiencing SUBD.

Recent findings suggest that online peer communities may offer similar benefits to face‐to‐face support.^15^ A qualitative systematic review considered how people with LTCs describe their experiences with online peer support. The main findings suggested that feelings of reciprocity, social support and access to experiential knowledge were experienced when accessing online peer support.^16^

To our knowledge, there have been no randomised controlled trials (RCTs) of online peer support and psychoeducation interventions available to people with a diverse range of LTCs and SUBD (i.e., platforms that are not condition‐specific). However, RCTs of face‐to‐face peer support were shown to be effective on mental and physical health outcomes for those with LTCs, including people with diabetes, asthma and cardiovascular disease.^17^, ^18^ Other research suggests peer support interventions for those currently experiencing depressive symptoms or higher scores of psychological distress were more effective at reducing depressive symptoms compared to usual care.^19^, ^20^

Online peer support platforms for varying health needs are abundant. Yet, there are no online peer support and psychoeducational interventions tailored to support those experiencing SUBD in the context of LTCs. This article is set within the context of a wider project aiming to develop an online peer support and psychoeducation platform for those living with LTCs and SUBD. Intervention mapping has been used to integrate theory and evidence and guide the development of the project.^21^ The study reported in this article is nested within the step ‘Intervention Mapping: Needs Assessment’.^21^

This article aims to explore the psychosocial needs of people living with LTCs and investigates their perspectives on online peer support interventions to inform their future design.

## 2 METHODS

### 2.1 Design

A focus group study of the psychosocial needs of people living with LTCs and their perspectives on online peer support.

### 2.2 Patient and public involvement (PPI)

This article is set within the context of a wider project that is developing an online peer support platform for those living with LTCs and SUBD. An intervention mapping^21^ and participatory, co‐production approach has been embedded throughout. Three groups were established as part of the participatory design as follows: (1) focus group participants; (2) a Research Advisory Group (RAG); and (3) a Participatory Design Panel (PDP).

The focus groups were formed of participants from King's College London's Integrated Care Consultation Partners Group (ICCPG), the Guy's and St Thomas' PPI group and the King's College Hospital PPI group. These groups bring together patients with physical/mental comorbidities and create a space for collaboration on research and public engagement at the mental‐physical interface.

A separate, independent RAG was formed of members also living with LTCs. They supported the study throughout by co‐producing all study documents and by collaborating on key decision‐making processes. They also co‐produced the focus group topic guide with the research team.

The PDP was made up of an external design agency, researchers, clinicians, a co‐applicant with lived experience (E. A.F.) and participants from the focus groups. The PDP will also be involved in the subsequently planned co‐design stages of developing the peer support platform.

### 2.3 Participants

Participants with LTCs were invited to take part in this study through flyer advertisements circulated through established PPI groups (the ICCPG, the Guy's and St Thomas' PPI group, the King's College Hospital PPI group) and through snowball sampling via these groups (e.g., word of mouth). Inclusion criteria were over 18 years of age, living with an LTC and the ability to give informed consent to participate. Exclusion criteria were insufficient English to be able to engage in focus group discussions. Participants were aware that they were being invited to discuss issues such as how their physical health condition affects their mental wellbeing and that the platform was being developed for use among people with SUBD and LTCs specifically. Three focus groups, with 10 people in total, were conducted, exploring the psychosocial needs of people living with LTCs and their perspectives on online peer support. Focus groups were intended to shift the experience of power from the researcher to the group of participants, and to enable participants to feel supported by the group and not isolated in their experiences.^22^

Due to restrictions imposed secondary to the 2019 novel coronavirus (COVID‐19) pandemic, focus groups were carried out online via videoconferencing platforms and group sizes were reduced due to the online shift. Consultations with the RAG and researchers with experience of online delivery of focus groups informed the choice of platform to ensure optimal engagement. Clear, standardised, step‐by‐step instructions were provided to participants on how to download, access and use the platform. All participants had the necessary equipment (i.e., a device to take part, a webcam and microphone) and were offered a practice call with a member of the research team before taking part. Full ethical approval was sought and granted by King's College London Research Ethics Office, PNM Research Ethics Subcommittee (HR‐19/20‐14938). Electronic informed consent was obtained from all participants before taking part in the focus groups. Participants were all reimbursed for their time and expertise.

### 2.4 Data collection

An experienced qualitative researcher (H. R.) facilitated the focus groups alongside co‐facilitators—Aymie Backler for two of the focus groups and Gabriella Bergin‐Cartwright for the final group. The co‐facilitator supported participants with technological difficulties and implemented a distress protocol if required, which was drafted due to the sensitive nature of the discussions.

The focus groups were designed to investigate the psychosocial needs of people living with LTCs and their perspectives on online peer support. The topic guide was co‐produced with members of the RAG, co‐investigator with lived experience and researchers.

It included open‐ended questions covering: the interaction of their physical and mental health, for example, ‘How does your health condition make you feel?’; efforts to access support, such as ‘Have you looked for information on living with a health condition?’; and what they might expect from using the platform, for example, ‘What concerns would you have about using this sort of online support platform?’ (Supporting Information: Appendix 1).

### 2.5 Analysis

Focus groups were audio recorded and transcribed verbatim. Transcripts were reread alongside listening to the audio recording to anonymise and check accuracy.

Reflexive thematic analysis (TA) was conducted (E. A.F. and H. R.) adopting a critical‐realist approach^23^, ^24^ and an inductive analysis methodology that sought to follow participants' priorities and concerns.^25^ This analysis was co‐produced using a participatory approach and therefore reflexive TA was selected by the authors as most appropriate due to its accessibility and acknowledgement that the authors play an active role in the analysis.^23^ The focus groups were not carried out in a social vacuum as our assumptions and experiences as researchers impact the research we conduct.^23^ HR (White British female, research assistant) is a source of support to family members living with various LTCs. EAF (White British female, communication strategist) lives with multiple LTCs (cystic fibrosis, cystic fibrosis‐related diabetes, adrenal insufficiency), and has carried out patient advocacy work for several years. EAF engaged with the research team in reflexive TA training.^23^ The highly relevant experiences of participants and depth of discussion enhanced the information power of this sample.^26^ Both authors (E. A.F. and H. R.) spent time independently reading and familiarising themselves with the transcripts and began to code and record key ideas from the transcripts. A process of member checking was also adopted by presenting an interim analysis of preliminary themes and codes to the PDP and, separately, the RAG. As all members of the PDP had participated in the initial focus groups, these interactive sessions offered a unique opportunity for post‐interview reflection. Feedback was sought on clarifying, developing and amending the final themes using the online collaborative tool Miro (© 2021 Miro). E. A.F. and H. R. individually coded the transcripts in consultation with the wider research team using Microsoft Excel. E. A.F. and H. R. then met regularly to discuss the data in detail to develop their initial interpretations and incorporate the feedback from the member checking work. The final generated themes are presented in Table 2.

### 2.6 Reporting

Reporting was guided by the Standards for Reporting Qualitative Research (SRQR), which consists of a 21‐item checklist.^27^ The SRQR has been used to ensure standards for presenting qualitative analysis are met, while also allowing the flexibility and approach of this work to be maintained.

## 3 RESULTS

### 3.1 Participant characteristics

Ten people with a range of LTCs participated across three online focus groups. Table 1 provides an overview of participant characteristics. The mean age was 57 (±11.4) years and 60% of those taking part in the focus groups identified as female. The majority (80%) of the participants used technology daily, and 30% had used internet support groups before.

**Table 1:** Description of participant characteristics.

	*n* (%)	Mean (±SD)	Range
Age^a^ (years)		57 (±11.4)	39–71
Female	6 (60)		
Male	3 (30)		
Ethnicity^a^			
Black British	2 (20)		
White British	6 (60)		
White European	1 (10)		
Do you have access to the internet at home?^a^			
Yes	9 (90)		
No			
How frequently do you use the internet?^a^			
Daily	8 (80)		
Weekly	1 (10)		
Monthly			
Never			
Other, please specify			
Have you used internet support groups before?^a^			
Yes	3 (30)		
No	6 (60)		
Long‐term conditions^a^			
Anaemia	1		
Arrhythmia	1		
Barrett's syndrome	1		
Chronic pain	4		
Compartment syndrome	1		
Dysphonia	1		
Emphysema	1		
Endometriosis	1		
Hypertension	1		
Irritable bowel syndrome	1		
Laryngopharyngeal reflux	1		
Lymphoedema	1		
Morton's neuroma	1		
Osteoarthritis	2		
Osteoporosis	1		
Peripheral polyneuropathy	1		
Rheumatoid arthritis	1		
Sciatica	1		
Scoliosis	1		
Severe allergic asthma	1		
Spondylolisthesis	1		
No response	1		
Number of participants living with multiple long‐term conditions^a^			
1 Long‐term condition	3		
2 Long‐term conditions	2		
3+ Long‐term conditions	4		

^a^ One participant did not provide characteristic information.

## 4 THEMES AND SUBTHEMES

Throughout the focus groups, a range of experiences were described in relation to the psychosocial needs of people living with LTCs and their perspectives on online peer support. We present three themes: (1) relationship between self and outside world; (2) past experiences of peer support; and (3) philosophy and vision of peer support. Table 2 provides an overview of the themes presented, corresponding subthemes, definitions and evidencing quotations.

**Table 2:** Themes and subthemes alongside quotations.

Theme	Subtheme	Subtheme description	Quotation
Relationship with self and the outside world	Mind–body separation	Participants express that healthcare culture generally tends to adopt the lens that both physical and mental care are separate.	When I was diagnosed, mental health issues didn't come into it. You had your condition and that was your condition. But now when we're asked to talk about how we feel … I find it really hard. (focus group 2, participant 2)
			(…) healthcare, um, practitioners, they'll just mention, okay, um, okay, what you're doing with your condition, how you're coping and you know, it's not how do you feel? And, and that so important to me, just asking that one simple question. (focus group 3, participant 1)
			(…) there's not much out there in terms of my physical condition and the impact that that has, you know, on my psychological well‐being (…) . (focus group 3, participant 1)
			Now I guess the culture doesn't really encourage that very much. There's this mind body separation. And also, I had to use the word spiritual, but I found a very good source to help me reflect and meditate and that's been enormously helpful. (focus group 1, participant 2)
			My mum was really tough with me. You didn't complain. You didn't cry. And she never let up on chores. (focus group 2, participant 2)
	Duality of health	When discussing their own health, both mind and body become entwined in the descriptions.	(…) physical health and, and, mental health colliding, um, because they, they both interlink with each other eventually. (focus group 3, participant 2)
			(…) my mental condition is something aside, but I think at some point the two did collide. (focus group 3, participant 1)
			I think I'll start with my physical health. Um, I've become fat, you know, staying indoors and lack of exercise, I've actually put on a lot of weight and it's impacted, um, the way I think or feel about myself in terms of, uh, wanting to go out. I'm thinking, I've put on so much weight and people are going to be looking at me saying, damn, you know. So, yeah, my confidence is a bit low when it comes to the weight issue. (focus group 1, participant 4)
	Predictable variability	Participants expect good and bad days with their health but the nature of when the bad days will occur is often uncertain.	But listening to the body and, listening to my body and finding out, um, when to take rests, when to get up and do something, how far to walk, uh, all those things. (focus group 1, participant 2)
			(…) my health has gone down to zero. I was on a scale of 100 and doing alright, I was coping on my own and then all of a sudden. (focus group 2, participant 2)
			(…) other people can make goals, long term goals and stuff but I just take each day as it comes. (focus group 3, participant 2)
			I overdo it on a good days and then have terrible days. (focus group 2, participant 2)
	Tension between self‐reliance and needing help	Wanting to be independent but also the discomfort with having to ask for help when support from others is needed.	I've asked for somebody's help to help me go upstairs, um, in, in the tube station to go through the stairs (…) And the person said, oh, I haven't got any money. (…) Can be tough on, on, on your mental health eventually. Because then you feel even more self‐conscious and anxious and, um … And, and, and paranoid in a lot of respect. (focus group 2, participant 3)
			Total strangers who are, like, loads older than me asking if they can help me which is extremely sweet but it makes me feel a bit pathetic. (focus group 2, participant 1)
			But the thing I, I've noticed the most in regard to mental health and that's sort of relationship within oneone's's self and the outside world, is, um, how would you say? The atmosphere, um, around one in the outer world, I find very unsettling. You know, the, the sort of vulnerabilities and the frailties and the suspicions and all these unsettling things, um, that seem to be within others, uh, affect me very deeply and I recoil. And it sort of re‐entrenches that, um, removal if you like, if that makes any sense. (focus group 1, participant 1)
			I struggle with asking for help. I have to have a mental breakdown and then someone says, let me help you, and that's when I'll allow it. (focus group 2, participant 2)
	Behind the mask	Often attempt to hide living with an LTC.	So I can't go into work, you know, feeling sick and looking sick and stuff. So it's, it's like there's two different me … Um, the sort of outward me and the inward me. It's actually quite exhausting. (focus group 3, participant 2)
			I've had people before that said, oh, you've got your makeup and stuff on. You don't look like you've got a problem with your back. And it's just how do you respond to that? (focus group 2, participant 4)
			It's difficult and, and you're in between and you try to hide as much as possible your disability, yet again because you don't want to be picked on, but obviously, you know, there's just not much you can do. (focus group 2, participant 3)
			But when it comes to relationship, it's a no‐go. Um, it's that fear that that person will run away. I've had that situation when someone realised what's wrong with me and they're like, oh, no I can't deal with that and stuff, and I always tend to hide things. (focus group 3, participant 2)
	Burden of increased self‐management	Changes to usual care during the pandemic have felt stressful.	Well the best you'd get is talking to your physio or your doctor by phone which isn't the same. (focus group 1, participant 3)
			I miss it. I really miss hydrotherapy. I really … Do. And no matter … I mean, tried to do it in the bath, but then you've got the … My … I'm on a meter. (focus group 2, participant 2)
			And everything has changed, um, I'm on a biologic. So I normally go into the hospital and they give me my injection, and now I have to learn within seconds, like how to do it myself. There wasn't any, um, demonstration of how to do this, um [sighs], so, yeah. It was really stressful. (focus group 3, participant 2)
Past experiences of peer support	Sharing knowledge and resources	Distributing health‐related information and experiences between peers is useful.	I think, again the element of peer support is more around, listen I've tried this and it's worked, or, I've heard someone that I know that has tried this and it has worked. (focus group 1, participant 4)
			Most powerful thing I've found is with the meet up groups, for example, on complex PTSD, um, it's being with other people who have similar experiences, and, um, there's a resonance there and just sharing resources and information. (focus group 1, participant 2)
			We'll have different discussions [in peer support group] about how, um, that impacted on them, you know, using that tool as well. And we might have slightly different experiences, but at least we know that it's something that works. (focus group 3, participant 2)
	A mutual validation	It was expressed that people with LTCs are best placed to understand how another person with a LTC may be feeling due to their personal experiences.	I've discovered that there are a few people out there who have the same issues that I do, um, so it's made me feel a little bit better. And with Facebook I've joined other groups, for example, with lung conditions like myself. And we're swapping ideas or I'm, not always contributing, but I'm reading and it does help in a way. (focus group 1, participant 3)
			I have a peer group for one of my long‐term conditions … we talk daily to each other, motivate each other, keep each other calm. (focus group 3, participant 2)
			(…) [my brother] he's, um, he gets very focussed, and he goes to the gym and he said to me, and he said, god, he said, if I had arthritis I would be having an operation within seconds. And it's a totally different attitude because what I've learned from the pain is extraordinary. (focus group 1, participant 2)
			The preparation [of going out] before and, and the sorting it all out afterwards is a nightmare, but I just really value the online stuff because, especially when it's a group … . (focus group 2, participant 4)
	Fear of negative reinforcement	Some participants may disengage or not engage at all with peer support platforms due to concerns around feeling worse after.	So I've not joined any online groups before because … Um, I don't know. I've just not felt that there was the right group for me. I think we spoke about, um, condition‐specific groups, and that really didn't help because everybody was comparing their back pain to your back pain and that just … Wasn't helpful. (focus group 2, participant 4)
			Cause I felt that [being a member of the Facebook peer support group], um, it was … Further, sort of underlining the fact that I did have, um, these conditions. And it just, I just sort of wanted to get away from it. And, you know, for a sense of normality. (focus group 3, participant 1)
			No size fits all. I think that sometimes a problem with, oh, well, we'll set up a peer group … And just assume that it's going to work and for everybody who's going to want to engage. (focus groups 3, participant 1)
Philosophy and vision of a peer support platform	A safe and credible zone	For peer support platforms to be a success they must be co‐produced, secure and a confidential space.	And if you're in a cocoon and there's only certain people that know the ins and outs of your life. You then become quite protective about what's going on. (focus group 2, participant 2)
			Talking about how mental health affects your pain, whatever that is, I think this is something new and it seems safe. Somehow, we can do it from our homes, we can listen to each other, but you haven't got to think about how to get somewhere. (focus group 2, participant 4)
			But I mean it's interesting. I'd be far more likely to use this because I think there's some, there's credibility behind it. (focus group 3, participant 1)
			Um, within my culture, it's like a taboo … When it comes to mental health. Um, so it's making the site, um … There, there's easy access to the sites where you don't need to go for a long process to kinda get to the stage. (focus group 3, participant 2)
	Reflect lived experiences	Peer support platforms need to consider personal differences and similarities of those using them and should reflect a space that they can all access.	That the peer support, um, or supportive or, uh, situations tend to be too structured and not reflecting the, the more authenticity of actual experience. (focus group 1 participant 1)
			So, yeah, I think, uh, an online peer support, um, forum, or a, uh, service or whatever yeah, you want to call it, might actually be very, very beneficial. Especially in these times that we've now realised that a lot of the services that people, or the support that people are being referred to quite, to be honest, inappropriate, uh, for their, for their needs. So yeah, especially I think on co‐morbidity, it's really quite difficult, um, to get the support you need. And you've got more than just one condition that you have to deal with. (focus group 1, participant 4)
			Absolutely. I think we have to, um, be very mindful of, um, [sighs] cultural sensitivity, and what is appropriate for one group might not be appropriate for another group … Culturally diverse references will increase engagement. (focus group 3, participant 1)
	Transparent motivations	Peer support platforms should not feel too prescriptive or corporatized.	And they frighten me terribly. I found them very presumptuous. Especially [name]. That was in such a structured, non experiential view um, it's, um, yes, it was, it was, um, quite contrived and synthetic. Um, and yeah. (focus group 1, participant 1)
			They're addressing business and businesses were talking. I mean, it's very good that people have more expansive sensitivities towards the mind, certainly, and I don't recoil from that, that's precious. But when it, things can get corrupted along the way by, um, um, scenario, other agendas, shall we just say. And it's very conspicuous in the commercial world I'm sure. But, but, in the sense it's a commercial gain to address it rather than the authenticity going to. You know, it's a completely different dynamic. (focus group 1 participant 1)
			That's another thing, actually, that I think is a benefit is that it's being run by [university name] [snapping sound] rather than a corporate entity or some even social enterprises, or even charities, that the, um, your ethics, at [university name], the ethics at [university name] are really, you know. (focus group 3, participant 1)
	Technology becomes an essential skill	Technological literacy is key due to the impact of remote living and working.	Well I've found, um, it's been a strangely positive experience in the way that, um, that, uh, I quite enjoy being on my own and it's given me a lot of time to reflect and to do a lot of Zooming around in different groups. (focus group 1, participant 2)
			And so since then [beginning of lockdown] I had, um, Zoom, uh like Microsoft Teams, uh, counselling sessions which I found a little awkward at times. Um, however, yeah they went well. Uh, so yeah, and, yeah. So yeah, you know, it was just weird at first having, uh, sessions, um, yeah. But, um, yeah. (focus group 1, participant 4)
			And trying to do the technology frustrates me because if I can't hear, if I can't see, or, uh, there's breaking up, and then I just throw myself outside and then I overdo it. (focus group 2, participant 2)

Abbreviation: LTC, long‐term condition.

## 5 RELATIONSHIP BETWEEN SELF AND THE OUTSIDE WORLD

### 5.1 Mind–body separation

Participants felt that healthcare culture generally groups physical and mental care as separate entities, even in the context of LTCs. This separation was felt in previous experiences of treatments received in healthcare environments, ‘when I was diagnosed, mental health issues didn't come into it. You had your condition and that was your condition. But now when we're asked to talk about how we feel … I find it really hard’ (focus group 2, participant 2), and was reflected in the way some participants viewed their own health: as two distinct halves of mental and physical. Participants showed awareness of the complex nature of health in certain contexts (e.g., social situations, in the workplace). Despite this, they reported health discussions with clinicians as seeming reductive and more two‐dimensional in nature, without acknowledgement from their doctor or nurse that their physical health status was likely to be affected by the condition of their mental health. The discussion of these interactions with clinicians was broad and varied according to participants. For some, the emotional side of living with an LTC was never discussed with their healthcare professional (HCP).

Participants reported that clinicians either did not discuss mental health issues and/or did not seem to consider themselves to be in an appropriate role to discuss them, though this was not the case for all. One participant reacted with surprise on the occasion their physical health consultant raised the topic of mental health without being prompted by the participant. Overall, participants considered joined‐up care of their LTC and mental health to be rare. The importance of the simple question ‘how are you feeling?’ in the context of a consultation was highlighted. The separation of mental and physical health was sometimes present outside of clinical contexts too. One participant recalled how despite feeling unwell when growing up, ‘you didn't complain, you didn't cry’ (focus group 2, participant 2) and their mother did not provide any emotional allowances for their health condition.

### 5.2 Duality of health

Despite the perceived separation of mind and body in the context of healthcare, when discussing their own health, participants' descriptions of both mind and body became entwined. During analysis, it was not possible to discern whether each participant considered mental distress as a distinct condition unrelated to their physical health, or distress as a direct result of their physical health. However, there was awareness of physical and mental health impacting on each other. The language used to describe this was striking: ‘my mental condition is something aside, but I think at some point the two did collide’ (focus group 3, participant 1), in reference to mental and physical health converging. In particular, there was an understanding of the role that lack of exercise or diet could have on mental wellbeing and physical health, ‘I've become fat, you know, staying indoors and lack of exercise, I've actually put on a lot of weight and it's impacted, um, the way I think or feel about myself’ (focus group 1, participant 4).

### 5.3 Predictable variability

This subtheme was strongly emphasised by participants and captures how participants expect to experience good and bad days with their health, but also find it hard to predict when the bad days will occur. This manifested in difficulties making plans and an attitude of ‘take each day as it comes’ (focus group 3, participant 2). Participants discussed the consequences of ‘overdoing it’ on good days, which subsequently led to bad days. Some demonstrated an awareness of how they might prevent a bad day, for example, taking preventative measures to alleviate physical limitations: ‘listening to my body … when to take rests … how far to walk’ (focus group 1, participant 2). For others, the onset of a bad day appeared suddenly without an obvious cause‐and‐effect relationship.

### 5.4 Tension between self‐reliance and needing help

This subtheme captures the discomfort that can come with living with an LTC in environments and locations that are physically difficult to access or participate in due to the physical limitation(s) of a health condition or disability. Some participants sought independence and consequently experienced discomfort when asking for help. Sometimes this discomfort was clearly evidenced, ‘I struggle with asking for help. I have to have a mental breakdown … and that's when I'll allow it’ (focus group 2, participant 2), for others it was implicit, ‘I've asked for somebody's help to go upstairs, um, in, in the tube station to go through the stairs … . And the person said, oh, I have not got any money … [which] can be tough on, your mental health’ (focus group 2, participant 3). It was clear that these interactions with members of the public caused distress.

### 5.5 Behind the mask

This subtheme refers to participants' occasional attempts to hide from others that they are living with an LTC. Some described how exhausting it can feel trying to conceal living with an LTC in the workplace, ‘so I can't go into work, you know, feeling sick and looking sick and stuff. So it's, it's like there's two different me … Um, the sort of outward me and the inward me. It's actually quite exhausting’ (focus group 3, participant 2), all the while receiving judgements from others on their appearance and perceived level of sickness. For example, participants received comments such as ‘you don't look like you've got a problem with your back’ (focus group 2, participant 4) in their work environment. In more personal settings, such as in a romantic relationship, judgements by a partner about their LTC had led to feelings of rejection and a desire to hide their LTC and full identity in future: ‘when it comes to relationships, it's a no‐go. Um, it's that fear that that person will run away’ (focus group 3, participant 2).

### 5.6 Burden of increased self‐management

This subtheme illustrates the varied impact that COVID‐19 lockdowns had on the treatment and management of LTCs. Most participants had experienced negative changes to both their self‐management and to the standard of clinical care that they usually received, which was described as stressful. Some experienced a lack of usual care and oversight from HCPs. This had a knock‐on effect of either increased self‐management to cope with symptoms, ‘so I normally go into the hospital and they give me my injection, and now I have to learn within seconds, like how to do it myself’ (focus group 3, participant 2), or an inability to manage a treatment because self‐management was not an option. Examples given were not restricted to pharmacological treatment, but also affected other types of treatment such as hydrotherapy for joint pain, which was not available during lockdown.

## 6 PAST EXPERIENCES OF PEER SUPPORT

### 6.1 Sharing knowledge and resources

Circulating health‐related information and experiences between peers was considered useful and a key reason for participating in peer support. The reasons given for sharing knowledge were manifold. One participant explained, ‘being with other people who have similar experiences, and, um, there's a resonance there and just sharing resources and information’ (focus group 1, participant 2). Other participants mentioned sharing what had worked for them personally and the enjoyment and optimism that came with showing proof of personal benefit. Interestingly, even if a resource had not benefited them personally, participants still enjoyed hearing about it, as evidence of success for another: ‘we might have slightly different experiences, but at least we know that it's something that works’ (focus group 3, participant 2).

### 6.2 Mutual validation

This subtheme captured the sense of recognition and affirmation participants reported when encountering someone with similar symptoms through peer support. It was expressed that people with LTCs are best placed to understand how another person with an LTC may be feeling due to their personal experiences. Simply the act of finding another with the same or similar symptoms could have this effect: ‘I've discovered that there are a few people out there who have the same issues that I do, um, so it's made me feel a little bit better’ (focus group 1, participant 3). For others, the sense of validation was found in the ongoing actions of peer support: ‘[…] we talk daily to each other, motivate each other, keep each other calm’ (focus group 3, participant 2).

### 6.3 Fear of negative reinforcement

There were not always positives to be found through shared experiences; for some participants, encountering people with similar symptoms made them feel worse. For this reason, they had chosen not to engage with peer support in the past. *Fear of negative reinforcement* encapsulated the feeling of hearing about negative health experiences from others and ‘[…] wanting to get away from it’ (focus group 3, participant 1). Two rationales for this were given. First, the conversation itself was perceived as negative or not solution‐focused, or second, it served as a reminder of the participants' own health when they did not want to focus on it. Traditionally, peer support in people with LTCs has been centred around a particular condition, but we found evidence that this approach did not work for everyone. Several participants described encountering attitudes of competitive comparison where symptoms were pitted against each other: ‘condition‐specific groups […] didn't help because everybody was comparing their back pain to your back pain and that just wasn't helpful’ (focus group 2, participant 4). Finally, while acknowledging that a condition‐specific approach could be successful for some, participants pointed out that ‘no size fits all’ (focus group 3, participant 1) and it was important not to assume a particular initiative could engage all those who wanted support.

## 7 PHILOSOPHY AND VISION OF PEER SUPPORT

### 7.1 A safe and credible zone

According to participants, successful peer support platforms should be a secure and confidential space and their development should involve co‐production with members of the patient group that they aim to cater for. The need for safety while accessing peer support was a key concern, although there were different definitions of what it meant to be safe in this context. We found that being in a safe space could mean, amongst other things, an expectation of privacy, shared standards of behaviour or code of conduct, an environment that appeared credible by promoting or following a code of conduct or standards of best practice, made visible to patients or service users. One participant described a desire for a closed or private space in relation to the sensitive nature of their health: ‘you're in a cocoon and there's only certain people that know the ins and outs of your life. You then become quite protective about what's going on’ (focus group 2, participant 2).

Interestingly, there was also an emphasis on accessibility to peer support, which in practice could result in a less private space, by virtue of online peer support being easy to find and participate in. The need for accessibility and privacy is concisely summed up here: ‘within my culture, it's like a taboo when it comes to mental health. So it's about making the site, […] easy to access’ (focus group 3, participant 2). The ease of participating in online peer support was also discussed: ‘we can do it from our homes, we can listen to each other, but you haven't got to think about how to get somewhere’ (focus group 2, participant 4).

### 7.2 Reflect lived experiences

Several aspects of lived experience were considered important in peer support interventions: individual circumstances, variations in the presentation of comorbidities and cultural diversity. The first factor is the perceived inability of existing peer support initiatives to meet the needs of those with co‐morbidity. Participants felt it was difficult to provide the right support for someone living with more than just one condition, and this could result in something 'inappropriate for their needs' (focus group 1, participant 4). Participants were sensitive to online environments, which they saw as generic or standardised in relation to their health needs. It was felt this resulted in support that did not reflect their lived experience and was therefore perceived as less helpful or applicable. This type of experience was considered inauthentic because 'situations [which] tend to be too structured are not reflecting the authenticity of actual experience' (focus group 1, participant 1). Third, several participants raised the importance of visible cultural and social diversity. It was felt that this evidence of diversity determined what was relevant and positively perceived by each user and could increase engagement with a peer support platform.

### 7.3 Transparent motivations

Participants believed that peer support platforms should not feel prescriptive or commercial in nature. One participant explained that it could depend on the motivations of the platform creators (whether commercial or academic) that result in an unwanted and prescriptive user experience: 'But things can get corrupted along the way by […] other agendas, shall we just say. And it's very conspicuous in the commercial world' (focus group 1, participant 1). It was felt that a commercial imperative was not inherently negative but considered likely to impact the integrity or values of a platform, which participants were acutely sensitive towards.

### 7.4 Technology becomes an essential skill

Participants described how adapting to an increase in digital technology during the COVID‐19 pandemic and having technological literacy was key to coping with the impacts of remote living and working. There was a mixture of positive and negative sentiments shown towards using technology. The transition from face‐to‐face activities to digital interfaces affected all spheres of living, ranging from remote counselling, consultation with HCPs, socialising, working and exercising. One participant described beginning to use the ubiquitous communication platform ‘*Zoom*’, as a positive because it provided the opportunity for additional reflection and socialising in different circles: ‘[…] a lot of time to reflect and to do a lot of Zooming around in different groups’ (focus group 1, participant 2). A downside to digital interactions became clear when equipment or technology operated suboptimally, leaving participants feeling frustrated.

## 8 DISCUSSION

This exploration of the psychosocial needs of people living with LTCs and their perspectives on online peer support further develops the understanding of these participants' experiences. Offering new insights that can inform the future design and implementation of online peer support and psychoeducation interventions for those living with LTCs and SUBD. Three overarching themes were detailed based on the participants' accounts of their experiences and needs: (1) relationship between self and outside world; (2) past experiences of peer support; and (3) philosophy and vision of peer support.

Although our participants were not formally assessed for SUBD, their experiences indicated difficulties with their mental health as a direct result of living with an LTC. Despite acknowledging their mental healthcare needs alongside their physical healthcare needs, participants did not experience integrated, coordinated care. There was a clear distinction between how health was conceptualised in a clinical context versus personal experiences and descriptions outside of this clinical context. This indicates that the nonintegrated nature of the clinical contexts in which people with LTCs engage does not align with their needs. People living with LTCs are less likely to access psychological interventions aiming to reduce depression.^28^, ^29^ As a result, services such as Improving Access to Psychological Therapy (IAPT) Pathway for People with Long‐term Physical Health Conditions and Medically Unexplained Symptoms are aiming to coordinate IAPT services, providing psychological therapies embedded in physical healthcare pathways.^28^ Integrating these services is imperative, but more consideration also needs to be placed on the importance of supporting those with SUBD to prevent the worsening of their mental health difficulties. This is where interventions such as online peer support and psychoeducation could play a potentially cost‐effective role.

The focus groups were conducted in the first six months of the COVID‐19 pandemic in the United Kingdom. The themes generated must be viewed in the context of the chronology they occurred in during the COVID‐19 pandemic and findings cannot be fully decoupled or extricated from the unique circumstances of the time. A recent article explored the experiences of service users with mental health difficulties during the COVID‐19 pandemic. They concluded that service users found changes to their usual mental healthcare worrying, particularly when these changes were not effectively communicated.^30^ Participants recognised that online peer support and psychoeducation did not require them to leave home and could therefore reduce the burden of self‐management by helping people to feel more connected and supported by others in similar situations. This links closely with Griffiths et al.,^18^ who found that a layperson‐led, self‐management programme for Bangladeshi adults with various LTCs led to significant improvement in self‐efficacy and self‐care behaviours when compared to usual care. This is also in line with the National Institute for Health and Care Excellence (NICE) report showing that digitally‐based health and behaviour change interventions can support people to increase their self‐management behaviours and improve their wellbeing.^9^ Again, although participants were not formally assessed for SUBD, many shared mental health challenges and expressed a need for increased psychosocial support to address these needs. The subthemes of ‘predictable variability’ and ‘behind the mask’ shed further light on the psychosocial difficulties and the commonalities in physical symptoms experienced by those with LTCs. The fatigue induced by attempts to conceal physical health conditions from others, in addition to fluctuations in physical health symptoms, were shared as common occurrences by people with LTCs. Providing more support for the self‐management of common experiences, such as these for people with LTCs, in addition to providing a platform to voice these shared experiences could improve the lives of people with LTCs who also experience SUBD, alleviating the strain on healthcare services, and ultimately preventing progression to MDD.

Participants expressed the view that peer support offers both an opportunity to share knowledge and resources and can provide a sense of mutual validation. They felt peers with similar experiences are best placed to understand their personal situation and provide valuable support. This chimes with the findings of a qualitative systematic review that considered how people with LTCs describe online peer support; key underpinning elements included reciprocity, social support and access to experiential knowledge.^16^

Whilst research assessing the efficacy of peer support for depression found that peer support interventions were more effective at reducing depressive symptoms compared to usual care,^19^ mitigating the potential adverse effects of online peer support is also key. Easton et al.^31^ suggest that further understanding potential adverse effects of online peer support is vital. Crucially, participants from the current article felt that online platforms must not have an over‐commercial look as this can feel unsafe to interact with, presumptuous and untrustworthy to use. Participants also expressed concerns surrounding possible negative interactions with other users, leading to wariness and potential disengagement. Previous research has also suggested that negative experiences of online peer support could be related to the impact of reading about other peoples' negative experiences.^32^ For online platforms to feel safer, they need to reflect users' experiences—tapping into the importance of the authenticity of lived experience and cultural diversity and must be carefully moderated. Overly defined environments feel unrelatable to people with lived experience and thus unhelpful. The more organic and flexible the space, the more usable it is. Participants expressed that central to this is co‐production, so that the people intended to use a service to steer its design and development. Robust data on adverse effects and safety are needed to better inform wide‐scale adoption within health systems.

Finally, pathways of referral to online peer support platforms also need careful exploration. This is especially pertinent in settings where integrated care and screening of mental health are not regularly practiced in secondary care. Alternative referral pathways might be required, such as through primary care practice and/or self‐referral pathways.

### 8.1 Strengths and limitations

This work is nested within a larger project aiming to develop an online peer support and psychoeducational platform for those living with LTCs and SUBD. This project adopts a theoretically driven intervention design using the Intervention Mapping Framework.^21^ This article provides evidence for the first step in the framework of identifying the needs of the group. The nature of adopting a Reflexive TA methodology and a participatory approach allows for flexibility and acknowledges the researchers' active roles in analyses.^23^ This is an important strength of this article, as an interim analysis was presented to two PPI groups and their feedback was used to develop the final analysis. This allowed us to develop our understanding of the data and check the resonance of experiences.^33^ The co‐investigator with lived experience (E. A.F.) also undertook the analysis and write‐up of this article alongside colleagues, further strengthening the interpretation and resonance of our work.

It should be noted that 80% of participants described their technology use as daily, demonstrating a limitation in the transferability of these findings among people with lower technology usage. Future work should therefore explore the potential barriers which may play a role in preventing access and usability of online peer support platforms, for example, digital competency and technology literacy.

While the majority of the participants used technology daily, over half had not used internet support groups before. There was quite a range in confidence with technology described upon recruitment and some participants required further technological support to take part in the focus group. Conducting the focus groups remotely facilitated the participation of people living with LTCs who can face physical barriers to attending in‐person research. To prevent a digital competency divide, tractable solutions should also be explored to ensure accessibility to online peer support for all those with LTCs. These solutions could include a dedicated onboarding process and perhaps assistance with acquiring digital tools where the individual does not have access to, or ownership of, the required technology. Most participants were actively engaged in research relating to the psychological and physical interface as some were recruited from well‐established PPI groups. To develop our understanding of the psychosocial needs of those living with LTCs, it would also be key to engage those that are less involved in research and those who have little or no access to technology, as they may have varying needs that are important to explore.

Additionally, future work should explore the potential role that HCPs may have in facilitating online peer support. Their role(s) may be multifaceted, from screening, referral and signposting, to moderating the platform and contributing to the psychoeducational material. Therefore, future work is needed to explore these potential roles and what people with LTCs would view as the most valuable role HCPs may play. Also, a limitation of this work that should be recognised is that we did not use a clinical measure to assess the mental health of participants, so the findings are not specific to those with SUBD. However, the recruitment flyer was framed under the title of ‘Online peer support for preventing depression in people with LTCs: focus groups’ and all people recruited to this study were aware that they were being invited to discuss issues such as how their physical health condition affects their mental wellbeing.

### 8.2 Future implications

This article provides the needs assessment element in the larger context of this body of work. The findings from this work will directly inform the development phases of an online peer support and psychoeducational platform for people with LTCs and SUBD. This work details the shared experiences of people with LTCs, highlighting the lack of integrated care available to address both physical and mental healthcare needs. This is an area of concerning unmet need as people living with LTCs recognise how their mental and physical health influence one another. Online peer support is accessible, inexpensive and scalable, and might offer a way of bridging the gap in psychosocial care for LTC patients. Intervening earlier could improve lives and reduce the burden of comorbid mental illness on families, the NHS and society. This is particularly important given the known increased risk people with LTCs have of developing MDD. The findings from the work will also inform the future vision and philosophy of platforms designed to help support the psychosocial needs of people with LTCs.

## 9 CONCLUSION

Adults living with a range of different LTCs expressed the potential benefits that online peer support may have on supporting their psychosocial needs. They also expressed potential concerns around negative engagement with online peer support, highlighted by their discussions that emphasised the importance of these spaces feeling safe. Based on the shared experience of those who took part in this work and the value of co‐production, careful, collaborative consideration is essential to develop the guiding principles of a future peer support platform, to explore potential moderation processes, and co‐produce a moderation policy. That participants expressed that any online peer support platform needs to be a safe and credible zone highlights the need for platforms to be co‐designed with the people that will ultimately use them to ensure this is a priority throughout. These findings evidence how important identifying needs in the pre‐intervention design stage is to promote a more purposeful intervention design that is user‐led.

## AUTHOR CONTRIBUTIONS

Initial conceptualisation, methodology and funding acquisition of this work was undertaken by Lauren Rayner, Matthew Hotopf, Alan Simpson and Vanessa Lawrence. Investigation was conducted by Lauren Rayner, Hannah Rowlands and Elly Aylwin‐Foster. Supervision, project administration and validation provided by Grace Lavelle and Lauren Rayner. Visualisation provided by Alan Simpson, Vanessa Lawrence and Grace Lavelle. Data curation and formal analysis were undertaken by Grace Lavelle, Hannah Rowlands, Elly Aylwin‐Foster and Vanessa Lawrence. Finally, initial draft writing was completed by Grace Lavelle, Hannah Rowlands, Elly Aylwin‐Foster and Vanessa Lawrence with the full authorship team reviewing and editing the final submitted manuscript (Hannah Rowlands, Elly Aylwin‐Foster, Matthew Hotopf, Lauren Rayner, Alan Simpson, Grace Lavelle and Vanessa Lawrence).

## CONFLICT OF INTEREST STATEMENT

The authors declare no conflict of interest.

## Supporting information

Supporting information.

## Data Availability

Pseudo anonymised participant data may be available for research purposes on request to the study authors, subject to approval.
