# Supplementary material for: A qualitative exploration of the psychosocial needs of people living with long‐term conditions and their perspectives on online peer support
Source: Health Expect. 2023 Jul 17;26(5):2075–88. doi: 10.1111/hex.13814 (PMC10485302; doi:10.1111/hex.13814)
Supplement: Supplementary file 1 — Supporting information. [file HEX-26--s001.docx]

**Appendix 1 – Focus Group Topic Guide**

| **Topics** | **Questions and prompts** |
| --- | --- |
| COVID-19 | 1. ***How have the events over the last month affected your daily life?***  - Physical health - Mental health - Support - Routine - Contact with others, support - Use of technology - Psychological well being - Any ways you have found to help you cope |
| Impact on life | 1. ***Thinking about what life was like before coronavirus…how did your physical health condition affect your life?***  - Physical health- how? - Mental health- how? - Social life- how? - Relationships- how? - Activities of daily living- how? - Anything help with that? |
| Mental wellbeing | 1. ***How does your health condition make you feel?***  - Have you ever found it difficult to cope? - Emotions? - Psychological wellbeing? - Negative? - Positive? - Why do you think it makes you feel like that? |
| Support | 1. ***Have you looked for information on living with a health condition?***  - **What sort of help/information/support did you access?** - Family/friends - Formal mental health support - GP - Hospital staff - Support groups - **If none, what prevented you accessing support?** - **Information online?** - If **NO**, could you tell us why? - What puts you off about going online for information or support related to your health and wellbeing? - If **YES**, what sort of information or support have you accessed online? - Internet support groups - Social media groups - NHS web-based information - Can you tell us a bit about why you went online to look for info and support? - Did you find it useful? |
| Interest in the platform | 1. ***We are hoping to put together a website that helps connect people with LTCs. Is it something that you think you would use?***  - Why would you use it? - What would you use it for? - When do you think you would use it? |
|  | 1. ***What would you want from a website?***  - What would you want it to do? - What features would you like to see? - What issues should it focus on? |
|  | 1. ***Do you think you would prefer to use it on a computer or on your mobile phone?***  - Why? - Examples? |
|  | 1. ***What concerns would you have about using this sort of online support platform?***  - Safety? - Anonymity? - Usability? |
